# pH‐Responsive Isoprenoid‐Antitumoral Polymer Conjugates for Superior Drug Loading via Self‐Assembly and Endosomal‐Targeted Anticancer Activity

**DOI:** 10.1002/cmdc.202500810

**Published:** 2026-01-31

**Authors:** Camilla Passi, Tobias Neu, Nicole Schneider‐Daum, Claus‐Michael Lehr, Marc Schneider, Sangeun Lee

**Affiliations:** ^1^ Department of Pharmacy Pharmaceutical Materials and Processing Saarland University Campus C4 1 66123 Saarbrücken Germany; ^2^ Department of Pharmacy Biopharmaceutics and Pharmaceutical Technology Saarland University Campus C4 1 66123 Saarbrücken Germany; ^3^ PharmaScienceHub (PSH) Saarland University Campus E2 1 66123 Saarbrücken Germany; ^4^ Helmholtz Institute of Pharmaceutical Research Saarland (HIPS) Helmholtz Centre for Infection Research (HZI) Campus E8 1 66123 Saarbrücken Germany

**Keywords:** antitumor agents, drug delivery, nanotechnology, pH‐responsive drug delivery, polymer‐drug conjugate

## Abstract

Polymer‐drug conjugates (PDCs) are a promising strategy to enhance the delivery of poorly soluble drugs, particularly in cancer therapy. By improving solubility and enabling site‐specific accumulation, PDCs minimize systemic toxicity while maximizing therapeutic efficacy. PDCs often employ stimuli‐responsive linkers, such as Schiff's bases, to achieve controlled drug release in tumor microenvironments or acidic intracellular compartments. In this study, we designed a novel PDC by conjugating the anticancer agent farnesal (Far) to ∈‐Poly‐L‐Lysine (PL) via an imine bond, without using additional linkers. PL is a natural, biodegradable, water‐soluble polymer with inherent anticancer properties, while Far is a hydrophobic isoprenoid with potent antitumor activity. The conjugate (Far‐PL) displayed pH‐responsive behavior, remaining stable at physiological pH but releasing drugs under acidic tumor (pH 6.5) and endosomal (pH 5.5) conditions. Far‐PL exhibited enhanced cytotoxicity against A549 lung cancer cells compared to its components alone, while showing reduced toxicity towards noncancerous cells (Arlo cells). The amphiphilicity allows the conjugate to self‐assemble into stable nanoparticles with a positive surface charge, narrow size distribution, and 100% drug content—clearly exceeding conventional nanoparticles (5–10 wt%). This effective PDC design demonstrates strong potential to maximize tumor‐selective activity while minimizing off‐target effects, offering a promising platform for future cancer therapeutics.

## Graphical abstract

Polymer‐drug conjugate (PDC) entirely composed of active compounds with pH‐responsive properties. The PDC can self‐assemble into nanoparticles for intracellular delivery and endosome‐targeted efficacy.

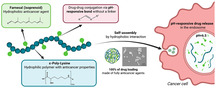

## 1 Introduction

Effective drug delivery remains a significant challenge across various therapeutic areas, including cancer, inflammation, and infectious diseases. In particular, hydrophobic drugs, such as doxorubicin, paclitaxel, prednisolone, and farnesol, often possess strong efficacy but suffer from instability and poor solubility, limiting their bioavailability and targeting.^[^ ^1^, ^2^, ^3^ ^]^ To overcome these limitations, polymer‐drug conjugates (PDCs) were first proposed in 1975 by Helmut Ringsdorf as a promising delivery system.^[^ ^4^ ^]^ PDCs offer distinct advantages, including the ability to tailor the physicochemical properties of drugs, improve pharmacokinetic and bioavailability, and enable controlled drug release mechanisms for targeted delivery.^[^ ^5^, ^6^, ^7^, ^8^, ^9^, ^10^, ^11^ ^]^ Since then, various PDCs utilizing polymer backbones, such as polyethylene glycol (PEG), polyethyleneimine (PEI), chitosan, dextran, poly(amidoamine) (PAMAM), and poly‐lysine, have been reported to enhance the delivery of a wide range of drugs, including doxorubicin, paclitaxel, and prednisolone.^[^ ^12^, ^13^, ^14^, ^15^, ^16^, ^17^, ^18^, ^19^ ^]^

The selection of the polymer backbone can affect the final efficacy of the system by improving pharmacokinetics, enhancing biocompatibility, and allowing high drug loading due to the availability of multiple functional groups for modification.^[^ ^5^ ^,^ ^20^ ^]^ Natural polymers are often chosen for PDC formulations because of their excellent biocompatibility and biodegradability, in addition to their biosafety and straightforward manufacturing processes.^[^ ^21^ ^,^ ^22^ ^]^ In particular, bioactive natural polymers, with chitosan and hyaluronic acid as interesting representatives of this group, are often used for the formulation of biocompatible PDCs.^[^ ^21^ ^]^ Chitosan exhibits a broad‐spectrum antimicrobial activity and has been used to conjugate numerous drugs, including paclitaxel. Paclitaxel conjugation on chitosan resulted in a new system for oral delivery of paclitaxel with comparable in vivo efficacy to the injected form but lower toxicity and improved retention.^[^ ^23^ ^]^ Similar to chitosan, hyaluronic acid is used to improve the solubility of drugs and confer tumor‐targeting capabilities. The conjugation of nimesulide, a potential anticancer drug, to hyaluronic acid resulted in a 1.47‐fold increase in tumor growth inhibition compared to the free drug.^[^ ^24^ ^]^ Although several PDCs successfully advanced to clinical trials, challenges such as suboptimal tumor accumulation, limited efficacy improvement, inappropriate drug release profiles, and rapid clearance remain significant hurdles.^[^ ^25^, ^26^, ^27^ ^]^

One strategy to overcome these limitations is the incorporation of stimuli‐responsive linkers in drug conjugation, enhancing the selectivity and efficacy of the PDCs. A stimuli‐responsive linker connects the polymer backbone and the drugs by covalent bonds, which are reversible or cleavable by triggering stimuli. By using such a linker for drug conjugation to the polymer backbone, selectivity and efficacy can be increased by targeted and controlled release, allowing the liberation of the drug in a time‐ and space‐controlled manner in response to specific triggers. Environmental pH changes can be an excellent trigger for stimuli‐responsive PDCs since numerous diseases, including cancers, present pH values altered from the physiological one.^[^ ^28^, ^29^, ^30^, ^31^, ^32^, ^33^, ^34^, ^35^ ^]^

In the field of pH‐responsive linkers, Schiff's bases, such as hydrazides, hydrazones, and imines, are suitable bonds due to their high sensitivity to acid‐catalyzed hydrolysis at pH levels below 6.5. This property makes them well‐suited for biological applications and is widely used for targeted drug release.^[^ ^36^, ^37^, ^38^ ^]^ A successful example of using the imine bond is the conjugation of doxorubicin (DOX) to dextran. By using imine bonds, the resulting PDC successfully achieved higher cellular uptake, greater efficacy, and decreased organ toxicity compared with the free DOX, owing to its controlled release.^[^ ^39^ ^]^

Another strategy to overcome the limitations of PDCs, such as insufficient accumulation and low stability, is nanoparticle formation. In addition to the ability of PDCs to protect the drug from degradation, nanoparticles can extend circulation time and enhance tumor accumulation through the enhanced permeability and retention (EPR) effect.^[^ ^20^ ^,^ ^40^ ^,^ ^41^ ^]^ Moreover, nanoparticles can further stabilize the conjugation between the polymer and drug in the PDCs. For example, when using stimuli‐responsive linkers that are unstable in aqueous environments, such as imines or boronic acids, nanoparticle assembly can embed these linkers in a more hydrophobic microenvironment. This limits water access, thereby improving the stability of the conjugate and enabling a more selective, acid‐responsive drug release strategy.^[^ ^42^ ^]^

Additionally, nanoparticles formed using PDCs result in higher drug loading compared to conventional nanoparticles, which typically achieve only 5–10 wt%. For example, conjugating DOX to PEG by imine bonds (PEG‐DOX) resulted in a 4.7‐fold increase in the loading of DOX (wt%) compared with the encapsulation of DOX in micelles formed with the diblock copolymer of PEG and an acid‐labile polycarbonate containing trimethoxybenzylidene acetals (PTMBPEC). Both systems were used for the co‐delivery of DOX and an additional anticancer drug, curcumin (Cur) or paclitaxel (PTX).^[^ ^43^ ^,^ ^44^ ^]^ PEG‐DOX‐Cur successfully resulted in 2.4‐fold increased loading (wt%) of the co‐delivered drug compared with the PTMBPEC system, efficiently co‐delivering DOX and Cur and achieving tumor‐targeted release. Another advantage of PDC‐nanoparticles is their enhanced cellular uptake compared to the free drug, as demonstrated by the development of self‐assembled nanoparticles formed by chitosan‐polylactic acid copolymer conjugated with curcumin (CS‐PLA‐CR).^[^ ^45^ ^]^ The intracellular uptake of CS‐PLA‐CR in the MCF‐7 cells after 12 h resulted in a 1.5‐fold higher drug internalization compared to the drug alone. These findings highlight that through strategic design and functionalization, PDCs represent a versatile and efficient platform for the controlled and targeted delivery of drugs.

In this study, we explored a PDC strategy utilizing ∈‐Poly‐L‐Lysine (PL) as a hydrophilic polymer backbone. PL is a biocompatible and FDA‐approved natural polymer.^[^ ^46^ ^]^ It is naturally produced by *Streptomyces albulus* with an average chain length of 25–35 L‐lysine residues, resulting in a molecular weight ranging from 3.5 to 4.5 kDa.^[^ ^47^ ^]^ The choice of using PL with a small molecular weight in this study is dictated not only by its natural production but also by its properties. PL with a number of residues around 20–30 is highly investigated for its anti‐tumor and antibacterial activity. Additionally, it displays beneficial characteristics such as water solubility, thermal stability, and absence of accumulation in organs or tissues, with an excretion time of 168 h, making it a suitable polymeric backbone for PDC.^[^ ^48^ ^,^ ^49^ ^]^ Furthermore, PL recently showed a preferential interaction, and thus toxicity, towards cancer cells, being able to inhibit proliferation and tumor angiogenesis.^[^ ^50^, ^51^, ^52^, ^53^, ^54^ ^]^ The anticancer mechanism of PL is not completely elucidated yet. However, it has been shown that the PL can interfere with the apoptosis pathway of the cell.^[^ ^55^, ^56^, ^57^ ^]^ Additionally, PL was proven to be able to stop tumor growth by lowering the levels of the cytokine vascular endothelial growth factor (VEGF), resulting in an antiangiogenic effect.^[^ ^55^ ^,^ ^58^ ^]^

Building on the concept of using a bioactive polymer to form a PDC system, we introduced in this study the hydrophobic anticancer agent, farnesal (Far). An antiangiogenic effect was reported for Far as well, which, together with other isoprenoids such as farnesol and geraniol, has been proven to inhibit tumor growth and selectively induce apoptosis in cancer cells.^[^ ^59^, ^60^, ^61^, ^62^, ^63^ ^]^ Moreover, the aldehyde group on Far can react with amines to form Schiff base linkages. This reaction offers pH‐responsive conjugation without the need for additional linkers, which is particularly advantageous for PDC applications. Conflicting with Far's high potential in cancer treatment are its physical and chemical properties, above all, its poor solubility and distinct instability.

Herein, to address these limitations, the hydrophobic Far was conjugated to the hydrophilic PL via Schiff's base (imine), resulting in a pH‐responsive amphiphilic PDC, termed Farnesal–Poly‐L‐Lysine (Far‐PL). The Far‐PL is expected to regenerate into bioactive compounds within the acidic compartments of cancer cells, thereby exerting anticancer effects. We therefore aimed to investigate its potential for controlled co‐release of the two components and cancer cell–specific efficacy, while preserving the advantages of each bioactive compound and overcoming their inherent limitations. In particular, considering the amphiphilic nature of the Far‐PL, we also examined its capacity to self‐assemble into nanoparticles, thereby demonstrating the feasibility of formulating PDCs composed entirely of natural bioactive compounds for practical therapeutic applications.

## 2 Results and Discussion

### 2.1 Synthesis of Farnesal‐Poly‐Lysine (Far‐PL)

**Figure 1:**
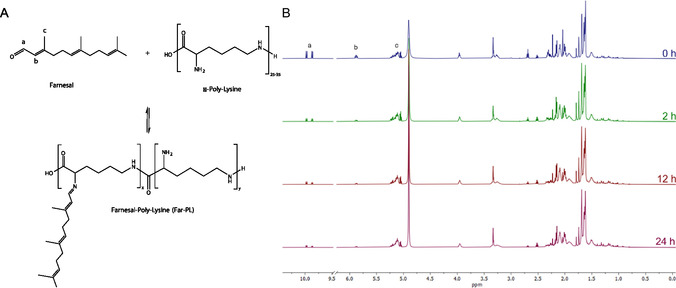
A) Synthesis scheme of Far‐PL starting from Far and PL. B) ^1^H‐NMR (500 MHz) spectra of Far‐PL100 (30 mg mL^−^^1^) during 24 h of synthesis. The peaks are marked.

The Far‐PLs were synthesized through a nucleophilic addition reaction between the aldehyde group of Far and the primary amino groups of PL (**Figure** **1**A). To investigate how varying degrees of substitution affect the properties of the PDC, we tested three different conjugation rates: 30, 50, and 100% (Far‐PL30, Far‐PL50, and Far‐PL100, respectively), by changing the molar ratio between the ‐NH_2_ groups of PL and Far (NH_2_:Far) in the reaction. The hydrophobicity of Far‐PL is expected to increase with a higher content of conjugated Far, resulting in changes in the PDC's behavior in degradation rate, NPs formation, and cytotoxicity.

Firstly, the synthesis was analyzed by performing FT‐IR measurement with Far, PL, and Far‐PL100. The resulting spectra show changes in the bonds after conjugation, indicating the successful modification of the starting compounds to form the final conjugate (Figure S1, Supporting Information). After this qualitative confirmation, we proceeded using quantitative analysis to further characterize the conjugate formation. We monitored the reaction using ^1^H‐NMR for 24 h. As shown in Figure 1B, after 24 h, the aldehyde proton peak (*t* = 0 h, a, *δ* ≈ 10 ppm) of the reaction mixture, 1:1 ratio of NH_2_ to Far (100% farnesylation, Far‐PL100), was depleted, proving the consumption of the aldehyde from Far. In the spectra, a decrease of the peak “b” (*δ* ≈ 6 ppm), connected to the vinylic proton on Far can be observed. This reduction is due to an increase in hydrophobic interaction, leading to peak broadening and thus decreased peak height.^[^ ^64^ ^,^ ^65^ ^]^ The conjugation rate was determined using q‐^1^H‐NMR by comparing the remaining aldehyde signal to the internal standard (maleic acid). The calculated conjugation rates were 95.3, 47.8, and 28.5% for Far‐PL100, Far‐PL50, and Far‐PL30, respectively. The results demonstrate the straightforward and adjustable synthesis of Far‐PL, indicating the ease of modulating individual properties such as hydrophobicity and obtaining a pH‐responsive system without the use of an additional linker for the conjugation.

### 2.2 Far‐PL pH‐Dependent Degradation

**Figure 2:**
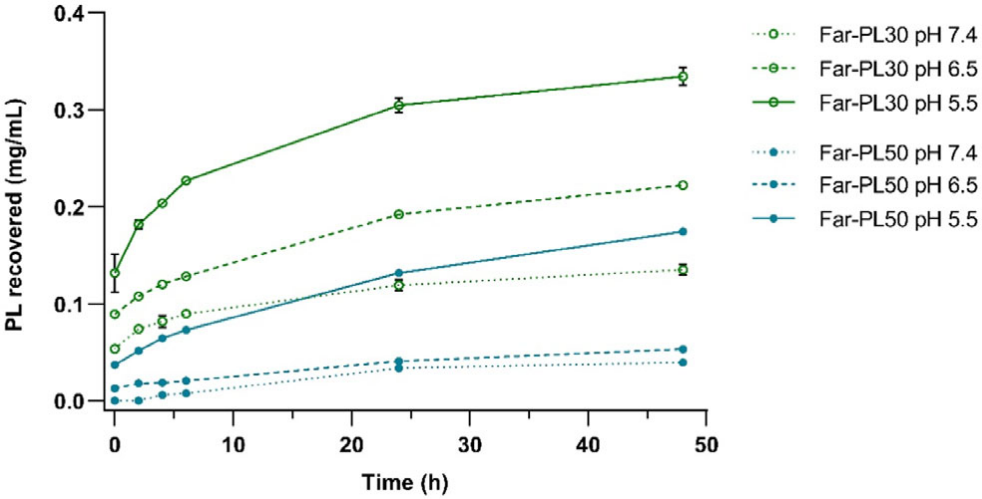
Far‐PL degradation at pH 7.4 (Tris buffer), 6.5 (MES buffer), and 5.5 (acetate buffer) during 48 h incubation at 37 °C. Degradation is expressed in terms of cumulative release of PL from Far‐PL30 and Far‐PL50 (5 mg mL^−^^1^). All data displayed are mean values ± standard deviation from *N* = 3 independent experiments.

After confirming the successful and controlled conjugation of Far on PL, we investigated the pH‐responsive cleavage of the imine bond and the restoration of free PL via the release of Far using HPLC. The Far‐PLs (Far‐PL30, Far‐PL500, and Far‐PL100) were treated at pH 6.5 and 5.5 to mimic the tumor microenvironment and the intracellular acidic compartments. Analysis at pH 7.4 served as a control to confirm their selectivity toward acidic conditions. As shown in **Figure** **2**, the release of PL from Far‐PL is influenced by both the pH and the degree of substitution.

Within 48 h at 37 °C, PL release from Far‐PL30 in acidic environments was accelerated, showing a 2.7‐fold increase at pH 5.5 and a 1.3‐fold increase at pH 6.5 compared to release at neutral pH (7.4). After treatment for 48 h at pH 5.5, up to 6.7% of free PL was recovered from Far‐PL30 and 3.5% from Far‐PL50. Note that, in this method, only free molecules of PL are quantified, with no Far remaining on any amino group.

Unfortunately, accurate quantification of Far released during incubation is technically challenging due to the high hydrophobicity, low solubility, and strong adsorptive nature of Far.^[^ ^66^, ^67^, ^68^ ^]^ However, in this study, the quantified pH‐responsive recovery of PL confirms the pH‐dependent release of Far, as the analysis accounts only for free PL molecules completely deconjugated from Far. Based on this, the detection of free PL in the samples indicates the presence of Far and allows estimation of its theoretical minimum release amount. Assuming an equal distribution of Far among the PL chains in the sample, we can calculate a release of 0.17 and 0.15 mg from Far‐PL30 and Far‐PL50, respectively, after 48 h of treatment at pH 5.5.

With a higher Far‐conjugation rate, a slower PL release profile at acidic pH, and enhanced stability at neutral pH were observed. The difference in the PL recovery between Far‐PL30 and Far‐PL50 is attributed to the higher hydrophobicity of Far‐PL50 due to a greater content of hydrophobic Far segments, leading to packing, reducing the accessibility of water to the imine bond, and so hindering its cleavage.^[^ ^38^ ^,^ ^69^, ^70^, ^71^, ^72^ ^]^ Additionally, a higher modification rate requires more time to reach a complete removal of Far from the PL backbone, resulting in a lower PL recovery rate. Thus, by tuning the Far‐conjugation rate and hydrophobicity of the polymers, the release of the active polymer and conjugated drug from the delivery system is controllable. Based on the results, a scenario can be anticipated in which the Far‐PL undergoes a first slight degradation in the tumor microenvironment and enhances the drug release upon tumor cellular uptake, leading to the improvement of the anticancer efficacy of the system.

The release of PL from Far‐PL100 after 48 h was also investigated, but no PL peak was detected (Figure S2, Supporting Information). The absence of a PL peak in Far‐PL100 suggests a slower release of Far, resulting in incomplete recovery of the PL chain fully free from Far over the 48 h analysis period. This supports the previous hypothesis: A higher conjugation rate limits a full recovery of PL, due to the limited accessibility of protons and water to the imine bonds. Based on these observations, Far‐PL100 was excluded from subsequent studies, anticancer activity and nanoparticle formation capabilities, due to its uncertain drug release profile and the hypothesized—later confirmed—lower anticancer activity of this conjugate within the experimental timeframe used in the study. However, the limited availability of pure PL from the Far‐PL100 does not imply an absence of Far release, as the PL release is the result of the complete release of Far from the conjugates. Moreover, the release profile under these experimental conditions does not directly represent the release kinetics under in vivo conditions. Far‐PL100, thus, may exhibit different potential under complex in vivo conditions where enzymes and proteins are present, which affect the release profile and stability of the conjugates.

### 2.3 Far‐PL Anticancer Activity

**Figure 3:**
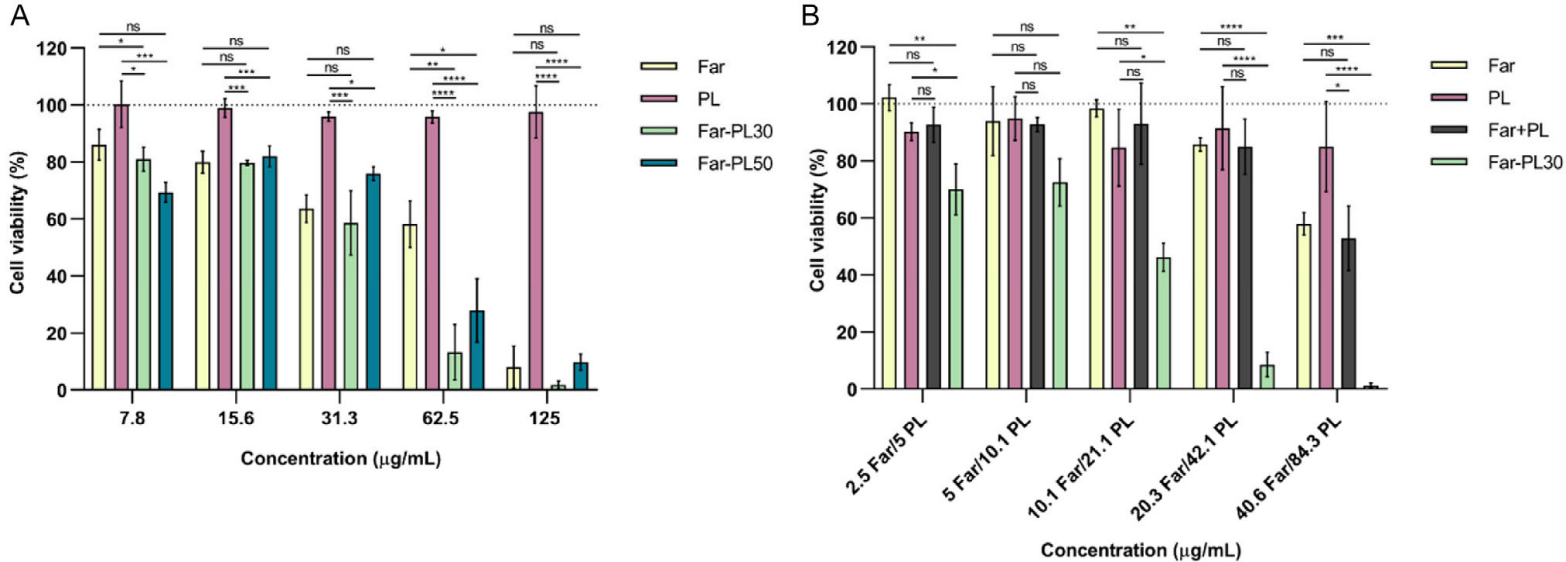
A) Cell viability of A549 cells analyzed by MTT assay after 24 h incubation with Far, PL, Far‐PL30, and Far‐PL50 at different concentrations (7.8–125 µg mL^−^^1^). B) Cell viability of A549 cells after 24 h incubation with Far‐PL30, and the equivalent amount of Far and PL, alone or in combination as a physical mixture. *p < 0.05, **p < 0.01, ***p < 0.001, ****p < 0.0001, ns, nonsignificant. All data displayed are mean values ± standard deviation from *n* = 9 (A), *n* = 6 (B), and *N* = 3 independent experiments.

Next, the efficacy of PL, Far, and Far‐PL was evaluated on A549 cells by performing a cell viability assay (MTT). Far showed a strong, concentration‐dependent cytotoxic effect on A549 cells (**Figure** **3**A). Cell viability decreased to ≈60, 40, and 10% at concentrations of 31.3, 62.5, and 125 µg mL^−^^1^ of Far, respectively. These results align with the previous reports describing isoprenoids such as farnesol to selectively inhibit tumor growth.^[^ ^62^ ^,^ ^73^ ^]^ Specifically, farnesol and its derivatives, including Far, have been proven to selectively induce apoptosis in different tumor cell lines.^[^ ^59^ ^,^ ^61^ ^,^ ^74^ ^]^

PL has been reported as a potential anticancer agent across various carcinoma cell lines, including HelaS3, HepG2, Caco‐2, and MCF‐7, by inducing apoptotic pathways selectively in cancer cells.^[^ ^51^, ^52^, ^53^ ^]^ However, no efficacy of the PL was observed in A549 cells within the tested concentration range. As shown in Figure 3A, after 24 h of incubation with PL in a concentration range from 7.8 to 125 µg mL^−1^, the metabolic activity of A549 cells remained above 90%. When PL is applied to A549, no interference with cellular pathways and toxicity is observed due to the poor permeation of the polymer alone into the cell (Figure S3, Supporting Information).^[^ ^75^ ^]^

Finally, the efficacy of Far‐PL has been investigated and compared with each component, PL, and Far. As shown in Figure 3A, the treatment of A549 with both conjugates, Far‐PL30 and Far‐PL50, resulted in a higher efficacy than for each compound alone. Far‐PL30 at 125 µg mL^−1^ reduced cell viability to less than 5%, which is significantly lower than the effect of Far or PL alone at the same concentration. At each concentration tested, the viability of cells treated with Far‐PL30 was lower than that of cells treated with Far or PL.

Comparing the conjugates, Far‐PL30 and Far‐PL50, Far‐PL30 showed higher efficacy up to 8‐fold at 125 µg mL^−1^. The higher efficacy of Far‐PL30 over Far‐PL50 can be explained by the higher release of PL and Far, connected to the different hydrophobicity of the polymers.^[^ ^72^ ^]^ Despite the higher content of Far in Far‐PL50, its hydrophobicity limits the release of the compounds, leading to a lower efficacy compared with the less hydrophobic Far‐PL30. An optimized release study of Far would be necessary to support this hypothesis and provide additional information on the effects of the compound in the intracellular environment.

For a more accurate comparison, the cytotoxicity of Far‐PL30 on A549 cells was compared with Far and PL by testing the single compounds at the same concentration in which they are present in Far‐PL30 (e.g., 125 µg mL^−1^ of Far‐PL30 is equivalent to 40.6 µg mL^−1^ of Far and 84.3 µg mL^−1^ of PL). In addition to testing Far and PL alone, the compounds were investigated by co‐adding them in the same well to observe the effect of their interaction without conjugation on cell viability.

As shown in Figure 3B, Far‐PL30 activity on A549 cells is significantly higher compared to the equivalent amount of Far and/or PL, resulting in a synergistic effect only when the two compounds are conjugated together. The improved efficacy of Far‐PL can be explained by the following reasons. First, as hypothesized, the conjugation of Far enhances the bioavailability of the hydrophobic Far by forming the amphiphilic Far‐PL, which helps overcome its solubility issues. Second, as aforementioned, the efficacy of PL is enhanced due to increased cellular uptake with amphiphilicity, enabling it to become active in A549 cells, even though PL alone was not effective on this cell line. Finally, the synergistic effect between Far and PL, once released inside the cell, contributes to the increased anticancer activity.

As aforementioned, Far and PL are reported to be able to induce apoptosis in cancer cells. This ability was evaluated by performing an apoptosis assay using Apopxin Green as an apoptosis marker. When apoptosis is triggered in cells, the first signal is the migration of phosphatidylserine (PS) on the outer leaflet of the cell membrane.^[^ ^76^ ^]^ By doing so, PS is exposed to the outer environment and can bind to markers like Apopxin Green, which can then be analyzed by fluorescence microscopy. The exposure of PS on the outer surface happens already in the early apoptosis stages, before morphological and metabolic changes can be detected. Together with Apopxin Green, cells have been stained with 7‐Amino actinomycin D (7‐AAD) and CytoCalcein Violet 450, markers for necrotic and living cells, respectively.^[^ ^77^ ^]^ Far‐PL30 and PL were applied to A549 cells and incubated for 2 h at a concentration of 62.5 µg mL^−1^, to allow the cells to interact with the compound without leading to complete death and excessive cell damage. Results (Figure S4, Supporting Information) indicate that in the presence of Far‐PL30, cells show clear signs of apoptosis and a loss of membrane integrity. When incubated with PL, cells appear alive (CytoCalcein Violet signal is present), but they show pre‐apoptotic events, with the Apopxin Green confined mostly to the cell membrane. The results confirm that PL has pro‐apoptotic activity in cancer cells; however, its low cellular permeability may hinder intracellular accumulation. Thereby, delaying apoptosis and reducing its apparent cytotoxic effect was shown in viability assays such as MTT. After conjugation with Far, Far‐PL30 showed enhanced efficacy on A549, decreasing the amount of living cells and showing apoptosis and morphological changes in the cell membrane even after a short incubation time. The number of necrotic cells was negligible both for PL and Far‐PL30.

### 2.4 pH‐Dependent Activation of Far‐PL in Endosomes

As discussed above, Far‐PLs possibly enhance the intracellular delivery of each anticancer component and improve their efficacy upon reaching acidic compartments due to the selective cleavage of the imine bond and the restoration of the drug structure. Therefore, we confirmed the cellular uptake of conjugates and subsequently assessed the recovery of drug efficacy within endosomal environments.

**Figure 4:**
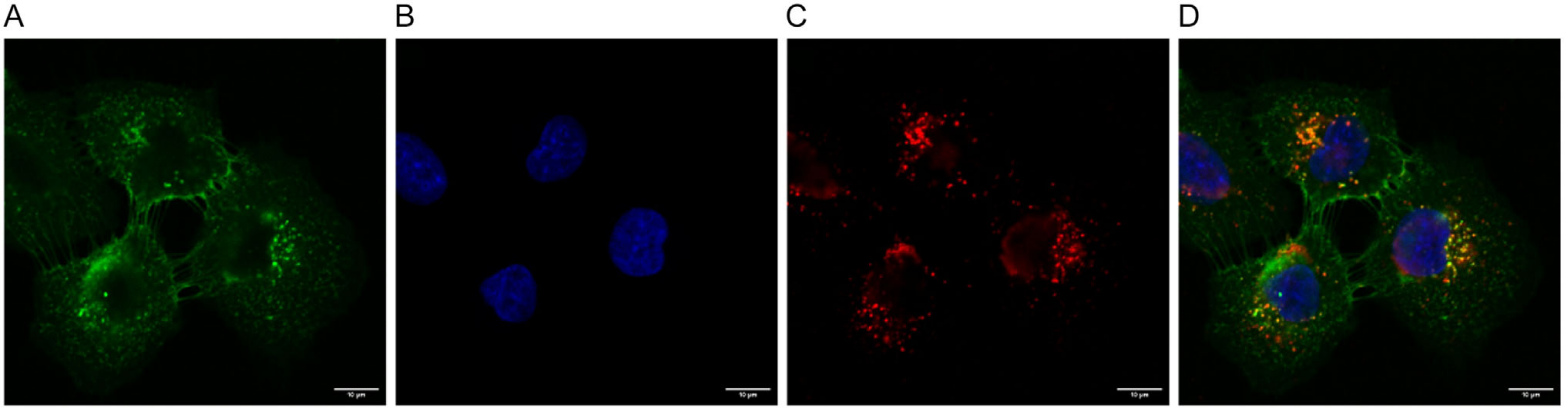
Far‐PL30 uptake in A549 cells after 4 h incubation with 10 µg mL^−^^1^ of compound. A) Concanavalin A AlexaFluor 488‐conjugate (cell membrane), B) DAPI (cell nucleus), C) Far‐PL30‐Rhodamine, and D) merged.

Rhodamine‐labeled Far‐PL30 (Far‐PL‐Rhod) was prepared, and its uptake in A549 cells was investigated by CLSM (**Figure** **4**). As shown in Figure 4, the Far‐PL‐Rhod was found inside the cells after 4 h. The temperature‐dependent uptake (Figure S5, Supporting Information) and the punctate intracellular distribution suggest the possibility that Far‐PL30‐Rhod effectively reached the intracellular environment via endocytosis and was placed in the endosomes or lysosomes.

Thus, we further confirmed the targeting of the polymer in acidified compartments indirectly by incubating A549 cells with a lysosomotropic agent, chloroquine (CQ), to inhibit endosomal acidification.^[^ ^78^ ^]^ Lysosomotropic agents are weak bases known for their ability to diffuse across the membrane of the vesicles and neutralize the acidic intracellular environments.^[^ ^79^, ^80^, ^81^ ^]^ Therefore, the addition of CQ would inhibit the pH drop in the endosome. Subsequently, if Far‐PL localizes in the endosomes, CQ would be expected to limit the acid‐triggered Far‐PL activation and, ultimately, reduce its efficacy.

**Figure 5:**
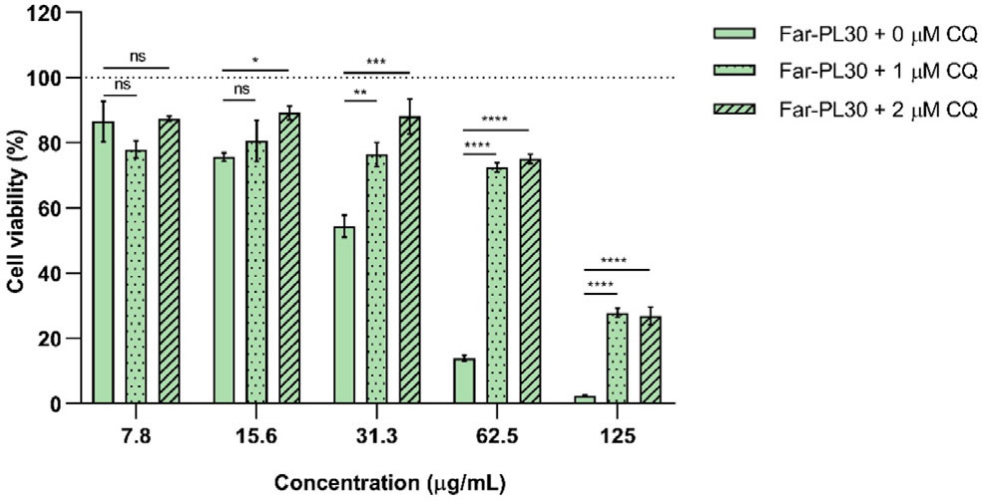
Effect of Far‐PL30 by chloroquine (CQ) concentration (0, 1, and 2 µM) on the cell viability, analyzed by MTT assay. A549 cells were incubated in CQ‐supplemented RPMI with 10% FCS before the Far‐PL30 incubation, followed by a further 24 h incubation with the Far‐PL30. *p < 0.05, **p < 0.01, ***p < 0.001, ****p < 0.0001, ns, non‐significant. All data displayed are mean values ± standard deviation from *n* = 9, *N* = 3 independent experiments.

CQ‐supplemented medium (1 and 2 µM, which results in negligible toxicity, Figure S6, Supporting Information) was used to treat A549 cells to investigate the impact of endosomal acidification on Far‐PL30 efficacy. As shown in **Figure** **5**, at the concentration of 125 µg mL^−1^ of Far‐PL30, the cell viability of A549 significantly increased from 5% for the untreated sample to 30% with CQ. In the same way, a stronger increase in cell viability is shown for 62.5 µg mL^−^^1^, where the viability increased from 15% without CQ to 70% with CQ. The difference in CQ concentrations had a minimal effect on the reduction of Far‐PL efficacy, indicating that in both cases, acidification was avoided. The results confirm that the activation of the Far‐PL is highly related to the acidification within the endosomes. When the acidification within the endosome was limited due to the lysosomotropic agent like CQ, the efficacy of the Far‐PL was reduced, which indirectly supports the acid‐triggered cleavage of the imine bond in Far‐PL and their activation in the endosomes. This also underlines the cellular uptake happening along endocytosis, reaching the endosomes.

### 2.5 Far‐PL Selectivity to Cancer Cells

After successful confirmation of Far‐PL efficacy on cancer cells, its activity was further tested on noncancerous cells to evaluate whether the polymer exhibits selective cytotoxicity. The evaluation of a drug's selectivity is an essential step in the development of novel drug candidates to assess their therapeutic safety and predict potential side effects.^[^ ^82^, ^83^, ^84^ ^]^ In this study, as a comparison to the A549 cell line, the immortalized monoclonal alveolar epithelial cells “Arlo” were selected to represent noncancerous lung tissue of the alveolar region. Arlo cells were produced via single‐cell printing, originating from the polyclonal human alveolar epithelium lentivirus‐immortalized cell line hAELVi.^[^ ^85^ ^]^ Arlo cells showed the ability to successfully replicate several physiological and functional features of the human respiratory system, additionally showing enhanced barrier properties.^[^ ^86^ ^]^ In vitro models such as Arlo are valuable tools for biopharmaceutical evaluation, enabling the investigation of drug properties, efficacy, and tissue‐specific responses in a physiologically relevant context.

**Figure 6:**
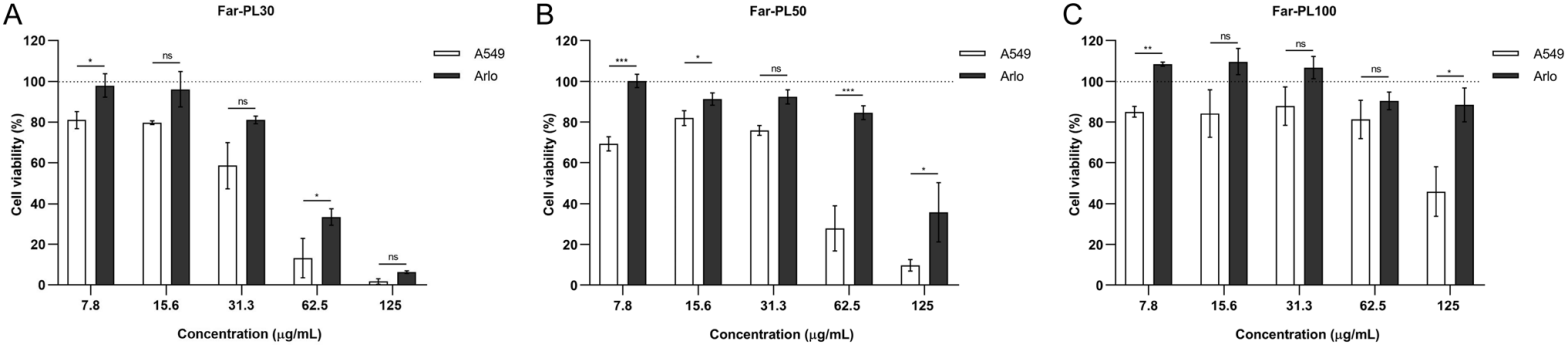
Cell viability of A549 and primary cell‐derived Arlo cells analyzed by MTT assay after 24 h incubation with A) Far‐PL30, B) Far‐PL50, and C) Far‐PL100 at different concentrations (7.8–125 µg mL^−^^1^). *p < 0.05, **p < 0.01, ***p < 0.001, ns, nonsignificant. All data displayed are mean values ± standard deviation from *n* = 9, *N* = 3 independent experiments.

Both cell lines were exposed to Far‐PL under the same experimental conditions, adjusting cell growth to better mimic cancerous and healthy tissues: A549 cells were grown until confluency in a 96‐well plate was reached (48 h); Arlo cells were cultured for 1 week after seeding them in a 96‐well plate to establish a stable barrier function to better mimic the epithelial lung tissue. As shown in **Figure** **6**, Far‐PL30 (A), Far‐PL50 (B), and Far‐PL100 (C) resulted in significantly less activity on Arlo compared with A549 cells. The higher cytotoxicity of Far‐PL30, compared with Far‐PL50 and Far‐PL100, is preserved across both cell lines. Far‐PL30 was cytotoxic to Arlo at concentrations above 31.3 µg mL^−1^, while Far‐PL50 and Far‐PL100 showed negligible toxicity, except for Far‐PL50 at 125 µg mL^−^^1^. These results revealed that Far‐PL shows a strong effect in A549 cells while exhibiting lower toxicity toward Arlo cells. The selective activity toward A549 can be attributed to several factors. First, the altered metabolic and membrane characteristics of cancer cells may facilitate the preferential uptake of the Far‐PL conjugate. For example, farnesol has been identified as an anticancer agent that can modulate various signal transduction pathways leading to apoptosis selectively in cancer cells, either by interfering with the mevalonate pathway, by altering phospholipase D signaling, or by regulating gene expression associated with endoplasmic reticulum (ER) stress response pathways.^[^ ^87^, ^88^, ^89^, ^90^ ^]^ Notably, in A549 cells, farnesol‐induced apoptosis is associated with a downregulation of HMG‐CoA reductase expression.^[^ ^91^ ^]^ Through such mechanisms, the conjugate may also have exhibited higher selectivity towards A549 cells. However, the intracellular mechanisms of PL in cancer cells remain poorly understood, and given the potential influence of conjugation on its effects, further mechanistic studies are needed to clarify its selectivity. Second, as discussed above about the Arlo cells, the enhanced barrier function and integrity of Arlo cells^[^ ^86^ ^]^—better mimicking in vivo epithelial conditions in the deep lung—may limit nonspecific uptake of the drug conjugate, thus reducing cytotoxic effects in healthy cells.

Furthermore, the selectivity observed in vitro may be more pronounced in vivo, derived from the enhanced permeability and retention (EPR) effect, due to Far‐PL's ability to self‐assemble into nanoparticles, thus potentially accumulates in tumor tissues. These findings provide a strong rationale for the continued development of Far‐PL and its formulation as a nanocarrier system, which will be explored in the following section.

### 2.6 Nanoparticle Formation

Nanoparticles have been widely explored for drug delivery due to their advantages in targeted delivery and prolonged circulation within biological systems, as well as their ability to enhance intracellular drug delivery via endocytosis.^[^ ^92^ ^,^ ^93^ ^]^ Despite these advantages, nanoparticles suffer from limited drug loading capacity (5–10% w/w).^[^ ^94^, ^95^, ^96^ ^]^

The strategy of self‐assembling PDCs is used for the delivery of anticancer drugs to maximize both intracellular drug delivery and drug loading. Numerous systems have been developed to enhance the intracellular delivery of potent drugs like doxorubicin (DOX) or paclitaxel (PTX). The conjugation of DOX to poly(ethylene glycol) (PEG) via reduction‐responsive disulfide bond (PEG–disulfide–DOX) resulted in a self‐assembling system able to improve DOX delivery to cancer cells. The system achieved 37.1% DOX loading into the prodrug and an effective release when in a reducing environment.^[^ ^97^ ^]^ A similar strategy was adopted for PTX with the formation of an amphiphilic poly(ethylene glycol)‐acetal‐paclitaxel (PEG‐acetal‐PTX) prodrug. The system showed self‐assembling ability, with a high loading of PTX (60.3%) and increased efficacy on HeLa cells compared to the free or encapsulated drug.^[^ ^98^ ^]^

Nanoparticle formation using Far‐PL could effectively address the limitations in drug loading typically associated with conventional nanoparticles. Specifically, thanks to the amphiphilic properties of Far‐PL, hydrophilic PL chain and hydrophobic Far segments, may facilitate the self‐assembling of nanoparticles without the use of inert polymers to form nanoparticles or chemical linkers. As a result, the nanoparticles can be entirely composed of active agents, resulting in 100% active compounds. Moreover, since Far‐PL is activated and its efficacy is enhanced in intracellular acidic environments such as endosomes, a more refined endosome‐targeted delivery strategy using nanoparticle formation could further improve its effectiveness.

Thus, we demonstrated the ability of Far‐PL to self‐assemble into nanoparticles. For the nanoparticle formation with Far‐PL (Far‐PL30 and Far‐PL50), three different nanoparticle formation methods were used. Self‐assembly (SA), nanoprecipitation (NA), and nanoemulsion (EM) were compared and analyzed using dynamic light scattering (DLS). Unlike the SA by thin film hydration, the latter two methods, NA and EM, include stabilizers (PVA) in the formulation, which may increase the stability of the resulting NPs while slightly reducing the drug loading at the same time.

**Figure 7:**
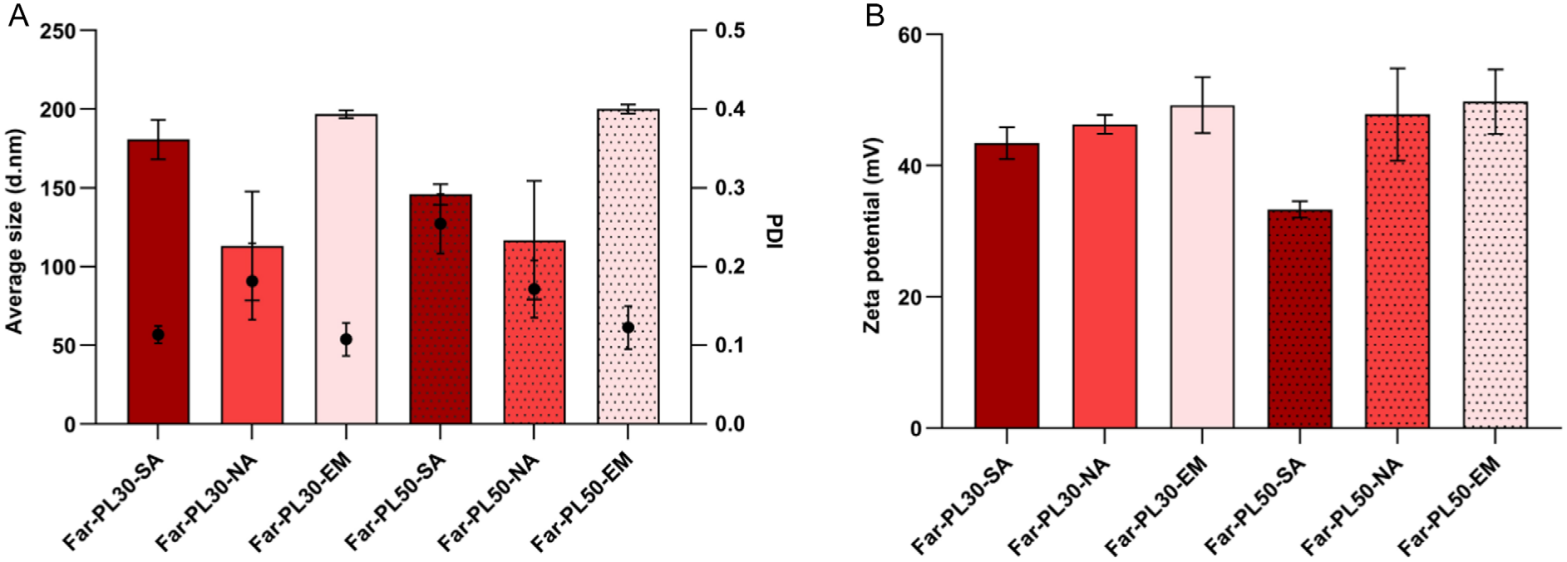
A) Hydrodynamic diameters (average size, d.nm) of nanoparticles (NPs) prepared using Far‐PL30 and Far‐PL50 with different methods: self‐assembly (SA), nanoprecipitation (NA), and nanoemulsion (EM). B) Zeta potential of NPs obtained from the different preparation methods. All data displayed are mean values ± standard deviation from *N* = 3 independent experiments.

**Figure 8:**
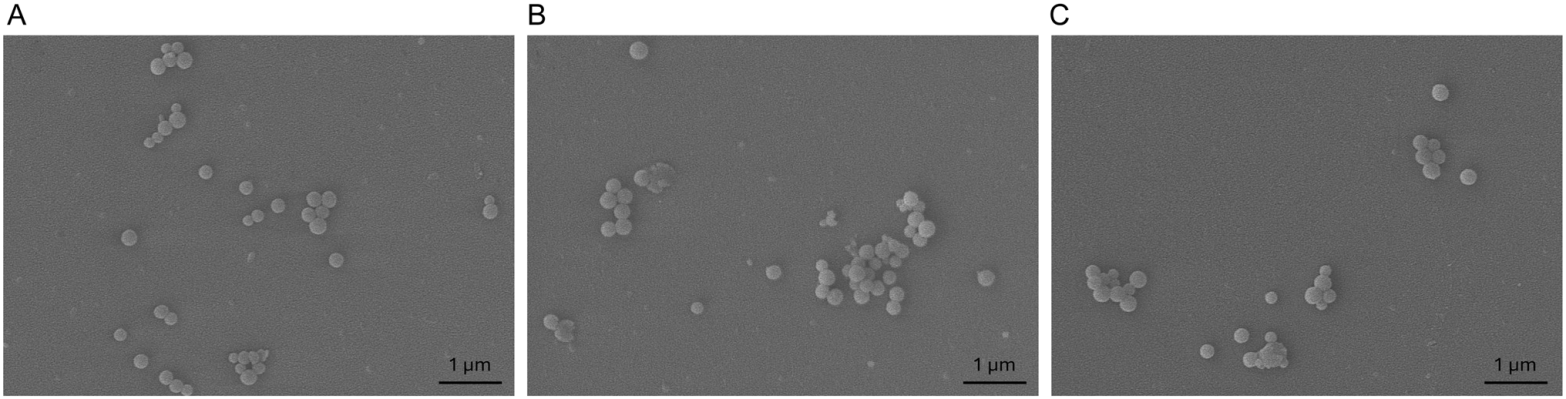
NPs morphology observed with SEM. NPs were formed by A) self‐assembling, B) nanoprecipitation, and C) nanoemulsion using Far‐PL30.

As shown in **Figure** **7**A, NPs resulted in different sizes based on the production method and on the conjugation rate of the polymer. NA‐NPs resulted in the smallest particles (*D*_H_ = ≈95–100 nm), both with Far‐PL30 and Far‐PL50, while for SA‐NPs and EM‐NPs, no trend in size was observed by methods (*D*_H_ = ≈150–200 nm). Overall, NPs showed an average size below 200 nm and PDI between 0.1 and 0.25 for each formulation, indicating that a colloidal suspension with a narrow size distribution was obtained.^[^ ^99^ ^,^ ^100^ ^]^ The particle size and dispersity observed by DLS were further confirmed through scanning electron microscopy (SEM), which allowed for the observation of the nanoparticle morphologies (**Figure** **8**). NPs formed with Far‐PL30 by self‐assembly (Figure 8A), nanoprecipitation (Figure 8B), and nanoemulsion (Figure 8C), all displayed a spherical shape with sizes ranging from 100 to 200 nm.

Additionally, NPs were characterized in terms of their surface charges by measuring their zeta potential (Figure 7B). Different from the NPs’ size, zeta potential showed no significant differences depending on the formulation methods. All methods resulted in a zeta potential higher than +30 mV, reaching +45 mV for NPs from NA and EM methods. A zeta potential greater than ±30 mV, as observed in this study, is usually associated with a stable NPs suspension, where the strong charge repulsion between NPs prevents agglomeration and supports their stability and dispersion.^[^ ^101^ ^,^ ^102^ ^]^

**Figure 9:**
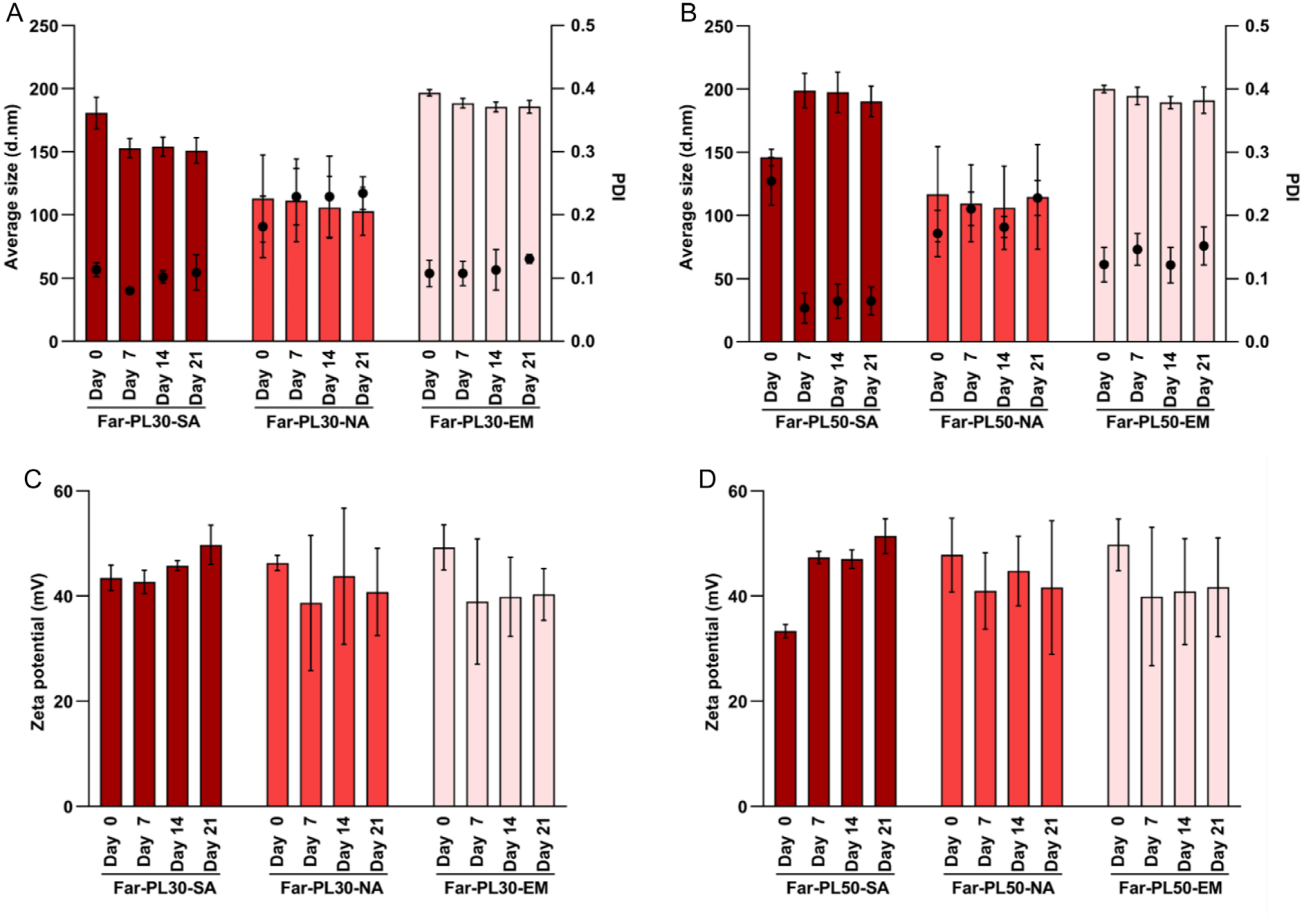
Average size and PDI from A) Far‐PL30‐NPs and B) Far‐PL50‐NPs. Zeta potential from C) Far‐PL30‐NPs and D) Far‐PL50‐NPs. NPs were stored for 21 days at room temperature and measured in Milli‐Q water. All data displayed are mean values ± standard deviation from *N* = 3 independent experiments.

In this context, not only is the short‐time stability of interest, but also the time the colloidal formulation conserves its properties. *D*_H_, PDI, and zeta potential were measured every 7 days over 21 days at room temperature. For Far‐PL30 (**Figure** **9**A) and Far‐PL50 (Figure 9B), no relevant changes in size and size distribution were observed, indicating NPs stability in aqueous media, with no formation of bigger aggregates or degradation. Additionally, the absence of significant changes in the zeta potential over 21 days (Figure 9C,D) further supports their excellent colloidal stability. Thus, all methods resulted in nanoparticles that were stable over at least 21 days when stored in Milli‐Q water at room temperature. Resultingly, the SA‐NPs own similar properties and stability over time to those produced by NA and EM, suggesting the use of a stabilizer in the NP formulation is not crucial in the case of Far‐PL to achieve stable NPs.

As a result, we successfully proved the ability of Far‐PL to self‐assemble into stable nanoparticles, composed of 100% active agents and comparable in size, stability and morphology to the ones obtained by traditional methods. Far‐PL self‐assembled nanoparticles allow overcoming the loading limitations of current nanoparticles, introducing a basically excipient‐free approach that maximizes drug content and therapeutic relevance.

### 2.7 Nanoparticles Anticancer Activity

Nanoparticles formed using Far‐PL conjugates have been characterized not only in terms of size and zeta potential, but additionally in terms of their functionality in comparison to the free polymer. We proceeded with analyzing the anticancer efficacy of nanoparticles formed with Far‐PL30 and Far‐PL50 using the previously mentioned methods.

**Figure 10:**
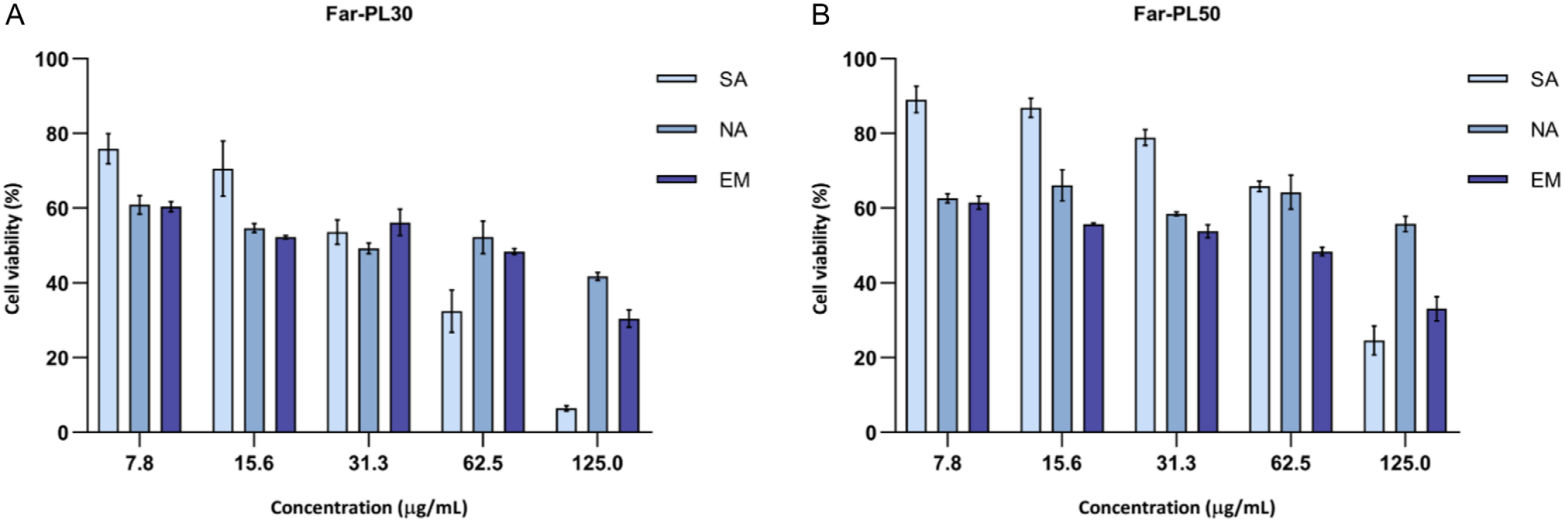
Cell viability of A549 cells analyzed by MTT assay after 24 h incubation with nanoparticles formulated using A) Far‐PL30 and B) Far‐PL50 at different concentrations (7.8–125 µg mL^−^^1^) and comparing three different production methods: self‐assembling (SA), nanoprecipitation (NA) and nanoemulsion (EM). All data displayed are mean values ± standard deviation from *n* = 9, *N* = 3 independent experiments.

As shown in **Figure** **10**, the conjugation rate dependent activity is retained after nanoparticle formation, with changes between the three different formulations. For the SA, the activity of the polymer is retained with no substantial changes compared with the free polymer. This primarily indicates that pH‐responsive drug release occurred successfully after NP formation, with release kinetics comparable to those of the polymer itself. However, under the cell experiment conditions employed, partial self‐assembly of the polymer cannot be prevented due to hydrophobic interactions, leading to the coexistence of polymeric and nanoparticulate forms. This coexistence may explain the observed similarity in efficacy between the SA and the polymer.

NPs formed by emulsification or precipitation showed slightly different efficacy compared with the polymer and SA. When compared with SA, NA and EM showed lower efficacy on the cells, due to the presence of stabilizers, which leads to a change in the total weight of the sample and the final content of polymer. For NA and EM, both in Far‐PL30 and Far‐PL50, higher efficacy was observed for EM than for NA, especially at higher concentrations (62.5 and 125 µg mL^−^^1^). This is due to the different formulations, which present a different stabilizer:polymer ratio. The starting stabilizer:polymer weight ratio in the nanoprecipitation method is doubled compared to that in the nanoemulsion method, leading to an overall lower content of polymer for the same weight of sample.

By observing the results from the assay, it is possible to conclude that the properties of the single polymers (Far‐PL30 and Far‐PL50) are retained after nanoparticle formation, with higher efficacy for lower conjugation rate, but the overall properties of the delivery system are changing based on the production method. Nanoparticle formation can offer the advantage of stabilizing the polymer, protecting it from early degradation, and resulting in increased cellular uptake. We believe that further studies on the advantages of nanoparticle formation using Far‐PL, in addition to the investigation regarding the drug release from the nanoparticles in comparison to the free polymer, can give useful insight into the pharmacokinetics of the system and provide an opportunity for tuning the drug release and the final anticancer activity by adjusting the production method.

## 3 Conclusion

In this study, we developed a pH‐responsive polymer‐drug conjugate (PDC) composed entirely of active agents, farnesal (Far) and ∈‐Poly‐L‐Lysine (PL), without the use of additional linkers, to obtain controlled release and maximize the efficacy of each component. The conjugation between Far and PL was highly efficient, with a coupling rate exceeding 95%, and the imine bond formed was selectively cleaved under acidic conditions. This pH‐responsive behavior facilitates targeted drug release within acidic endosomal compartments of tumor cells. Indeed, the activity of Far‐PL was significantly higher compared to when each active ingredient was administered individually at higher concentrations. In particular, when the endosomal pH was not maintained acidic by chloroquine, the activity of Far‐PL was reduced, confirming experimentally that the enhanced activity of Far‐PL resulted from efficient cellular uptake via endocytosis and drug release in acidic endosomes. Additionally, Far‐PL showed selective efficacy toward cancer cells while showing minimal cytotoxicity in noncancer cells. Far‐PL has, furthermore, demonstrated the ability to self‐assemble into highly stable nanoparticles, hinting at its potential as a promising drug delivery strategy for targeting endosomes. Notably, since the particles are entirely composed of anticancer agents, this approach could address the limited drug‐loading efficiency of existing nanoparticle carriers. Therefore, with further verification of the therapeutic efficacy of the formed particles, the pH‐dependent targeted activity and nanoparticle formation of Far‐PL hold significant promise in cancer treatment.

## 4 Experimental Section

4.1

4.1.1

##### Materials

∈‐Poly‐L‐Lysine HCl (Average MW 3500–4500 Da) was obtained from Biosynth Carbosynth (Bratislava, Slovakia). Farnesal (mixture of isomers), Mowiol 4–88 (Poly(vinyl alcohol) Mw ≈ 31,000), and chloroquine diphosphate salt were purchased from Sigma–Aldrich (Steinheim, Germany). Deuterated methanol (methanol d4, 99.80% D) for NMR use was obtained from Eurisotop (Saint‐Aubin, France). Trifluoroacetic acid (TFA) (99+%, for HPLC) was purchased from Fisher Scientific (Schwerte, Germany). Maleic acid (standard for quantitative NMR, TraceCERT), LiChrospher 100 RP‐18 (5 µm) HPLC column, and Amicon Ultra‐0.5 centrifugal filters (100 kDa MWCO) were purchased from Sigma–Aldrich (Steinheim, Germany). For cell cultivation, RPMI‐1640 medium, trypsin/EDTA, Dulbecco's phosphate buffered saline, 3‐(4,5‐dimethylthiazol‐2‐yl)−2,5‐diphenyltetrazolium bromide (MTT), and Triton X were purchased from Sigma–Aldrich (Steinheim, Germany). NHS‐Rhodamine, 4′, 6‐diamidino‐2‐phenylindole (DAPI), and Alexa Fluor 488‐conjugate of Concanavalin A were purchased from Thermo Fisher Scientific (Darmstadt, Germany). Apoptosis/Necrosis Assay Kit (blue, green, red) was purchased from Abcam (Cambridge, UK). All the reagents were purchased with HPLC or analytical grade and used without any further purification.

##### Synthesis of Farnesal‐Poly‐Lysine (Far‐PL)

For the synthesis of Farnesal‐Poly‐Lysine (Far‐PL), 20 mg of PL was dissolved in 1 mL of methanol. To synthesize Far‐PL100, 38.4 µL of Far (1.57 × 10–4 mol, 1:1 molar ratio of NH_2_ on the PL and Far) was added to the PL solution and was left stirring for 24 h at room temperature. Far‐PL with different conjugation rates (50% for Far‐PL50 and 30% for Far‐PL30) between Far and PL were synthesized by adjusting the amount of Far to the NH_2_ in the repeating units of PL to obtain NH_2_:Far ratios from 1:1 to 1:0.5 and 1:0.3.

The synthesized Far‐PLs were analyzed using PerkinElmer Spectrum 400 FT‐IR and Bruker Ultrashield 500 NMR Magnet (500 MHz) in methanol‐d4 (MeOD). To quantify the conjugation rate between Far and PL, maleic acid was used as an internal standard quantifying the unreacted aldehydes of Far.

##### pH‐Dependent Degradation of Far‐PL

To investigate the degradation of Far‐PL at acidic pH, the Far‐PLs were incubated in different solutions simulating the physiological conditions (pH 7.4), the tumor microenvironment (pH 6.5), and the acidic intracellular compartments (pH 5.5).^[^ ^103^ ^]^ The polymers were dissolved in the treating solution (5 mg mL^−^^1^) composed of 75% buffer (0.1 M acetate buffer at pH 5.5, MES buffer at pH 6.5, or Tris buffer at pH 7.4) and 25% methanol. Methanol was included primarily for analytical purposes. Due to the limitations associated with farnesal, like its instability, pronounced hydrophobicity, and strong tendency to escape the aqueous phase, we introduced methanol to allow and facilitate its dissolution following release from the polymer. The polymers were incubated at different pH at 37 °C for 48 h with gentle shaking and were analyzed at predetermined time points: 2, 4, 6, 24, and 48 h.

To quantify the release of PL from the polymer, Thermo Scientific Vanquish HPLC was used, equipped with a LiChrospher 100 RP‐18 (5 µm) column. A gradient method composed of two eluents was developed: solution A (90% Milli‐Q water, 10% acetonitrile, 1 mL TFA) and solution B (90% acetonitrile, 10% Milli‐Q water, 1 mL TFA). For each treating solution, a calibration curve with pure PL was prepared. All experiments were performed in triplicate.

##### Cell Cultivation

For the investigation of Far‐PL anticancer activity, two cell lines were used in this study.

A549 cells (human adenocarcinoma alveolar epithelial cells) were obtained from DSMZ (Braunschweig, Germany, No: ACC 107) and were cultured in RPMI‐1640 medium (Sigma–Aldrich, Steinheim, Germany) supplemented with 10% fetal calf serum (FCS; Gibco, Waltham, MA, USA) and incubated in a humidified incubator under 5% CO_2_ at 37 °C. Cells were cultured in T75 cm^2^ flasks, with medium replacement every other day, and subcultured once per week. For the passage, cells were washed once with PBS (Sigma–Aldrich), detached using 3 mL of trypsin‐EDTA (Gibco) for 5 min at 37 °C and 5% CO_2_, and centrifuged at 4000 g for 4 min. After centrifugation, the cell pellet was resuspended with 10 mL RPMI, and the resulting suspension was used to count cells and prepare a new T75 cm^2^ flask with 2 × 10^5^ cells.

Arlo is a monoclonal human alveolar epithelial cell line developed using precision single‐cell printing to obtain a versatile in vitro model for biopharmaceutical research. Cells were purchased from InSCREENex (Braunschweig, Germany, INS‐CI‐1031). The cells were cultured in Small Airway Growth Medium (SAGM) Bullet kit (Lonza, Basel, Switzerland) supplemented with 1% (v/v) FCS (Gibco, 10270106) and 1% (v/v) penicillin‐streptomycin (Gibco, 15140122) and maintained in a humidified incubator at 37 °C with 5% CO_2_. For routine cultivation in T25 cm^2^ flasks, the culture medium was replaced every other day, and cells were passaged once per week. Therefore, cells were washed twice with PBS (Sigma–Aldrich, D8537), detached using 1.8 mL of trypsin‐EDTA (Gibco, 25300054) for 6 min at 37 °C and 5% CO_2_, and centrifuged at 300 g for 4 min. The resulting cell pellet was resuspended with 5 mL SAGM, and the cell suspension was used to prepare a new T25 cm^2^ flask with 0.7 × 10^6^ cells. Prior to seeding, flasks were coated with 2 mL of a coating solution containing 1% (v/v) Fibronectin (1 mg mL^−^^1^, Corning, 356008, Corning, NY, USA) and 1% (v/v) Collagen (3 mg mL^−1^, Sigma–Aldrich, C4243) in sterile distilled water. Coating was performed for 1 h at 37 °C and 5% CO_2_.

##### Cell Viability

Cell viability in response to Far, PL, Far‐PL, and nanoparticles was assessed by performing an MTT assay on A549 cells. For the assay, cells were seeded (2 × 10^4^ cells/well) in a 96‐well plate and grown for 48 h. The samples were diluted from the stock solution with the culture medium to the final concentrations (0, 7.8, 15.6, 31.3, 62.5, and 125 µg mL^−1^) and incubated for 24 h. For the experiment, the medium was supplemented with 0.1% DMSO to allow Far dilution without altering cell viability. As a positive control, 2% Triton‐X was applied. After 24 h, cells were washed with PBS and incubated with 10% MTT reagent. After 4 h incubation with MTT reagent, the medium was aspirated, and DMSO was added, followed by incubation for 20 min. The absorbance of formed formazan was measured by Tecan plate reader (InfiniteM200, Tecan, Männedorf, Switzerland) at 550 nm.

##### Far‐PL Apoptotic Effect on A549 Cells

To investigate the ability of the conjugate to induce apoptosis in cancer cells, an apoptosis/necrosis assay was performed. For the assay, cells were seeded (2 × 10^4^ cells/well) in an 8‐well plate and grown for 48 h. Far‐PL30 and PL were diluted from the stock solution with the culture medium to the final concentrations of 62.5 µg mL^−1^ and incubated with the cells for 2 h. Afterwards, cells were washed twice with 100 µL of Assay Buffer and incubated for the staining with 200 µL of Assay Buffer containing 2 µL of Apopxin Green, 1 µL of 7‐AAD, and 1 µL of CytoCalcein Violet 450 for 45 min at room temperature. After the staining, cells were washed twice with 100 µL of Assay Buffer, and finally, 200 µL of the buffer were added. Cells were imaged using confocal laser scanning microscopy (CLSM) (LSM710, Zeiss, Oberkochen, Germany) with a 40× water immersion objective. For the visualization of fluorescent dyes, DAPI, FITC, and Rhodamine channels were used. Images were analyzed with Fiji software (version 2.16.0).

##### Far‐PL Activation in the Endosomes

To confirm the endosomal‐targeting properties of Far‐PL, an MTT assay with a lysosomotropic agent was performed. Chloroquine diphosphate salt (CQ) was used as a lysosomotropic agent to inhibit endosomal acidification and observe the changes in Far‐PL efficacy in cell viability.^[^ ^78^ ^]^ A549 cells were seeded (2 × 10^4^ cells/well) in a 96‐well plate and grown for 48 h in the incubator in RPMI with 10% FCS. After washing with prewarmed PBS, the medium was replaced with medium supplemented with different concentrations of CQ (0, 1, and 2 µM) and incubated for 3 h. Far‐PL (0, 7.8, 15.6, 31.3, 62.5, and 125 µg mL^−1^) with CQ (0, 1, and 2 µM) were added to the cells. After 24 h incubation, the MTT assay was performed as previously described.

##### Far‐PL Selectivity to Cancer Cells

Far‐PL selective activity on cancer cells was investigated by testing the polymer on the Arlo monoclonal human alveolar epithelial cell line, used as a control, compared with the A549 cells. For the assay, cells were seeded at a density of 3.3 × 10^4^ cells/well in 96‐well plates previously coated with 100 µL of the fibronectin/collagen solution for 1 h under the same incubation conditions. After seeding, cells were cultured for 7 days, with medium changes every 2 days. Before the assay, samples were diluted from the stock solution with the culture medium (supplemented with 0.1% DMSO) to the final concentrations (0, 7.8, 15.6, 31.3, 62.5, and 125 µg mL^−1^) and incubated for 24 h. Afterward, the MTT assay was performed as previously described for A549 cells.

##### Cellular Internalization of Far‐PL

The cellular uptake of Far‐PL was tested on the A549 cell line. Far‐PL was previously labeled using NHS‐Rhodamine (Far‐PL30‐Rhod). For the labeling, Far‐PL30 (2.72 mg) and NHS‐Rhodamine (7 × 10^4^ mmol) were dissolved in methanol. The solution was left stirring overnight and washed using a dialysis membrane (Tubing Spectra/Por 7, 3.5 kDa MWCO, Sigma–Aldrich, Steinheim, Germany) in Milli‐Q water for 72 h.

Cells were seeded (2 × 10^4^ cells/well) in an 8‐well imaging chamber and grown for 48 h. Afterward, cells were treated with the Far‐PL30‐Rhod (10 µg mL^−1^) for 4 h. After washing the cells with prewarmed PBS twice, the cells were fixed with 4% paraformaldehyde for 30 min at room temperature. Nuclei were stained with DAPI (0.2 µg mL^−1^), and the cell membranes were stained using Alexa Fluor488‐conjugate of Concanavalin A (50 µg mL^−1^). Cell uptake of Far‐PL30‐Rhod was visualized by CLSM using a 63× water immersion objective. DAPI, Alexa Fluor 488, and NHS‐Rhodamine were excited at 405, 488, and 561 nm using an argon laser, and the emission was recorded at 410–507, 496–603 and 566–703 nm, respectively. Images were analyzed with Fiji software.

##### Preparation of Far‐PL Nanoparticles

The ability of Far‐PL to form nanoparticles (NPs) was observed by preparing nanoparticles using three different methods.

Self‐assembly (SA): Thin film hydration method was used by modification of the developed protocol.^[^ ^104^ ^]^ Briefly, Far‐PL30 (150 µL, 26.6 mg mL^−1^) and Far‐PL50 (130 µL, 30.7 mg mL^−1^) in methanol were transferred separately into a round‐bottom flask, and the methanol was removed using Rotavapor (R‐100, Buchi, Essen, Germany) to obtain a thin polymeric film. Afterward, Milli‐Q water (0.5 mL) was added while gently shaking the flask to allow nanoparticle formation.

Nanoprecipitation (NA): Far‐PL in methanol (100 µL, 30 mg mL^−1^) was diluted with 900 µL of acetone and added dropwise to 1% polyvinyl alcohol (PVA) aqueous solution (2 mL). The solution was left under mild stirring overnight to allow the evaporation of the organic solvents.

Nanoemulsion (EM): Far‐PL in methanol (160 µL, 30 mg mL^−1^) was diluted with 640 µL of ethyl acetate and added to 1.6 mL of 1% PVA solution. The solution was stirred for 10 min before forming the emulsion using an ultrasonic homogenizer (Bandelin Sonopuls HD 3100, Bandelin, Berlin, Germany) for 30 s at an amplitude of 37%. Afterward, 1 mL of Milli‐Q water was added, and the solution was left under mild stirring overnight to remove organic solvents.

The resulting NPs prepared with SA, NA, and NE methods (SA‐NPs, NA‐NPs, and EM‐NPs, respectively) were purified using Amicon Ultra‐0.5 centrifugal filter (100 kDa MWCO) at 5000 g, for three cycles of 10 min each, at 20 °C. Finally, the NPs suspension was characterized and freeze‐dried for quantification.

##### Characterization of Far‐PL‐NPs

Hydrodynamic diameter (*D*_H_), polydispersity index (PDI), and zeta potential of NPs were measured using Zetasizer Ultra (Malvern Panalytical, Malvern, UK). Samples were diluted with Milli‐Q water in a concentration range from 0.8 to 1 mg mL^−1^ and placed in a folded cuvette for zeta potential measurement. NPs’ stability in Milli‐Q water was investigated by monitoring the *D*_H_, PDI, and zeta potential over 3 weeks. The NPs stock solution was kept at room temperature and diluted with Milli‐Q water for the measurement. The measurements were performed in triplicate.

Morphology was visualized using scanning electron microscopy (SEM, Zeiss EVO HD15, Carl Zeiss, Oberkochen, Germany) at 5 kV and 20,000x magnification under high vacuum. SA‐NPs, NA‐NPs, and EM‐NPs prepared from Far‐PL30 were washed and placed on a silica wafer, air‐dried, and, for better imaging, sputter‐coated with a 10 nm gold layer, using Quorum Q150R ES sputter coater (Quorum Technologies Ltd, East Grinstead, UK) with a thin gold layer before the measurement.

##### Statistical Analysis

The data are presented as mean ± standard deviation (SD) based on independent experiments. For the statistical analysis, a one‐way analysis of variance (ANOVA) with a Bonferroni post hoc test was used. Statistical analysis was conducted using GraphPad Prism software.

## Conflict of Interest

The authors declare no conflict of interest.

## Author Contributions

**Camilla Passi**: data curation (lead); investigation (lead); methodology (equal); formal analysis (lead); software (lead); validation (lead); writing—original draft preparation (lead). **Tobias Neu**: investigation (equal); data curation (equal). **Nicole Schneider‐Daum**: supervision (supporting); conceptualization (supporting). **Claus‐Michael Lehr**: supervision (supporting). **Marc Schneider**: conceptualization (equal); methodology (equal); project administration (equal); supervision (equal); writing—review editing (equal). **Sangeun Lee**: methodology (equal); project administration (lead); supervision (lead); writing—review and editing (lead).

## Supporting information

Supplementary Material

## Data Availability

The data that support the findings of this study are available from the corresponding author upon reasonable request.
