# Supplementary material for: pH‐Responsive Isoprenoid‐Antitumoral Polymer Conjugates for Superior Drug Loading via Self‐Assembly and Endosomal‐Targeted Anticancer Activity
Source: ChemMedChem. 2026 Jan 31;21(2):e202500810. doi: 10.1002/cmdc.202500810 (PMC12860526; doi:10.1002/cmdc.202500810)
Supplement: Supplementary file 1 — Supplementary Material [file CMDC-21-e202500810-s001.pdf]

RESEARCH ARTICLE

pH-Responsive Isoprenoid-Antitumoral Polymer Conjugates for Superior Drug Loading via Self-assembly and Endosomal-Targeted Anticancer Activity

Camilla Passi [a,b,c], Tobias Neu [c,d], Nicole Schneider-Daum [d], Claus-Michael Lehr [c,d], Marc Schneider [b,c,d,*], Sangeun Lee [a,c,d,*]

[a] C. Passi, Jun.-Prof. Dr. S. Lee
Department of Pharmacy, Pharmaceutical Materials and Processing
Saarland University
Campus C4.1, 66123 Saarbrücken, Germany
E-mail: sangeun.lee@uni.saarland.de

[b] C. Passi, Prof. Dr. M. Schneider
Department of Pharmacy, Biopharmaceutics and Pharmaceutical Technology
Saarland University
Campus C4.1, 66123 Saarbrücken, Germany
E-mail: marc.schneider@uni.saarland.de

[c] C. Passi, T. Neu, Prof. Dr. C.M. Lehr, Prof. Dr. M. Schneider, Jun.-Prof. Dr. S. Lee
PharmaScienceHub (PSH)
66123 Saarbrücken, Germany

[d] T. Neu, Prof. Dr. C.M. Lehr, Dr. N. Schneider-Daum
Helmholtz Institute of Pharmaceutical Research Saarland (HIPS)
Helmholtz Centre for Infection Research (HZI)
Campus E8.1, 66123 Saarbrücken, Germany

Supporting information

WILEY-VCH

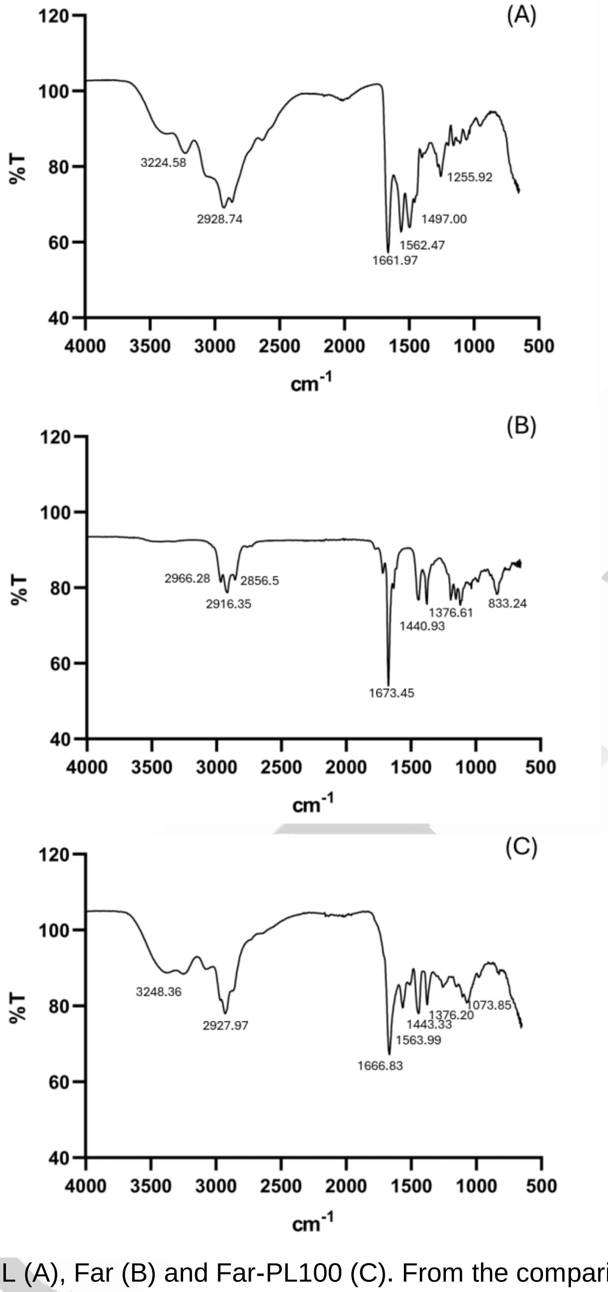

Figure S1 FT-IR spectra from PL (A), Far (B) and Far-PL100 (C). From the comparison of the three spectra, it is possible to observe how changes are occurring after the conjugation. In Far-PL30 spectrum (C) the peak area around 3500 to 2500 cm^{-1} shows modification due to the decrease of free amino groups in the molecule and the area around 1600 to 1400 presents modification connected to the formation of the new imine bond between Far and PL. Peak assignation: (A) N-H stretching (3224.58 cm-1); O-H stretching (2928.74 cm-1); C=O stretching (1661.97 cm-1); C-H bending (1532.47,1497 cm-1); C-N stretching (1255.95 cm-1). (B) C-H stretching (2966.28-2856.5 cm-1); C=O stretching (1673.45 cm-1); C-H bending (1440.93, 1376.61 cm-1); C=C bending (833.24 cm-1). (C) N-H stretching (3248.36 cm-1); O-H, C-H stretching (2927.97 cm-1); C=N stretching (1666.83 cm-1), C-H bending (1563.99-1376.20 cm-1), C-N stretching (1073.85 cm-1).

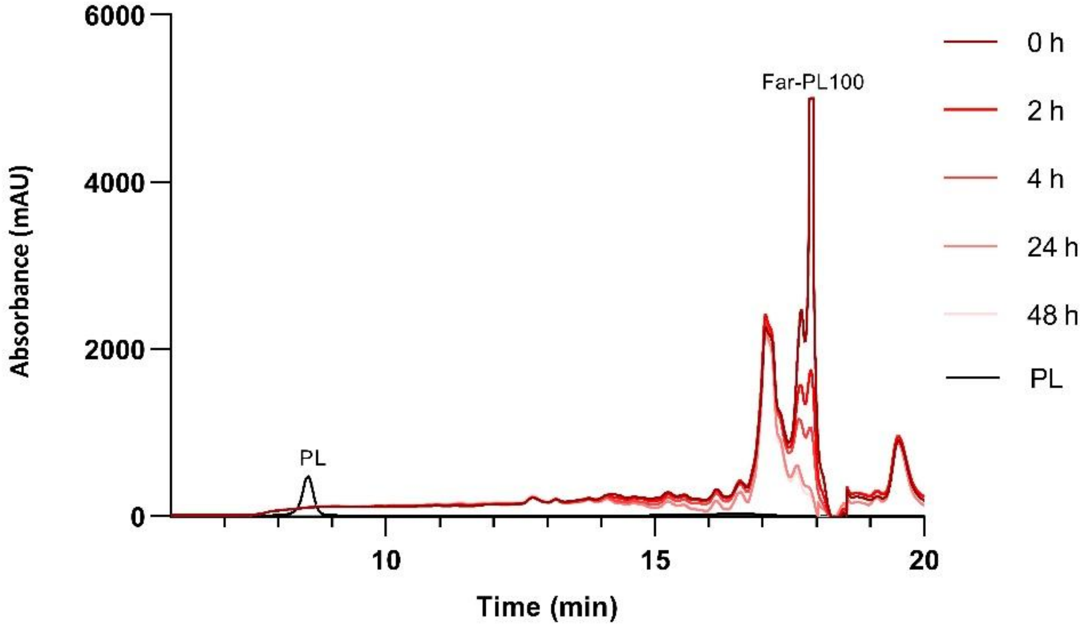

Figure S2 PL recovery from Far-PL 100 at pH 5.5 over 48 h at 37 °C, monitored by HPLC. As reference, pure PL was analyzed at the same conditions, showing a characteristic peak at 8.5 min. The absence of the PL peak in the chromatograms from Far-PL100 indicates no recovery of the polymer after 48 h of incubation. The peak at 17 min can be attributed to a small amount of Far in the solution, reaching its solubility limit in the 25% MeOH used for the analysis, hence not increasing over time. The peak at 18 min shows the decrease of FarPL100 in the sample, indicating that the cleavage of Far-PL bond is happening. As result, an undefined number of Far-PL conjugates with varying amount of Far still conjugated to PL is present in the sample, resulting from Far-PL100 undergoing different degrees of degradation. The decrease of the peak provides only qualitative evidence of Far release by showing reduced amount of Far-PL, but cannot be used to quantify the Far amount released.

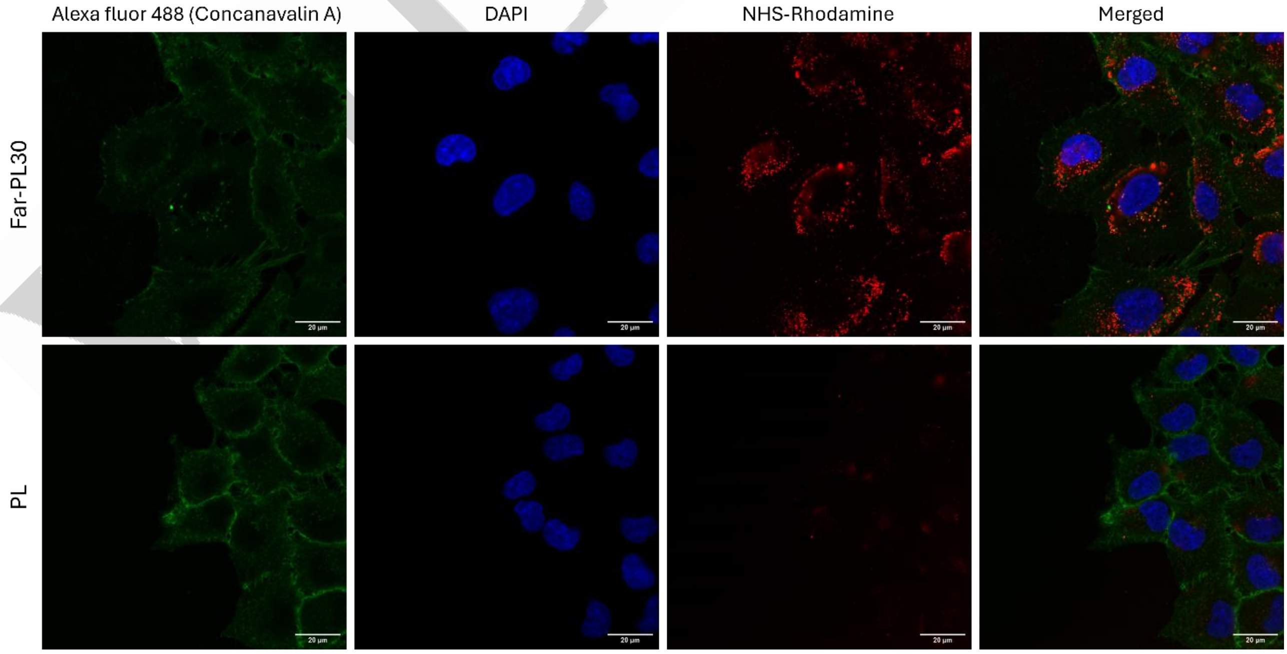

Figure S3 Cellular uptake of Far-PL30 and PL on A549 after 4 h incubation. It is obvious that the pure PL is not taken up into the cells, whereas the FarPL30 shows uptake in all cells in the image. The fluorescence intensity of labeled polymers was normalized prior to imaging.

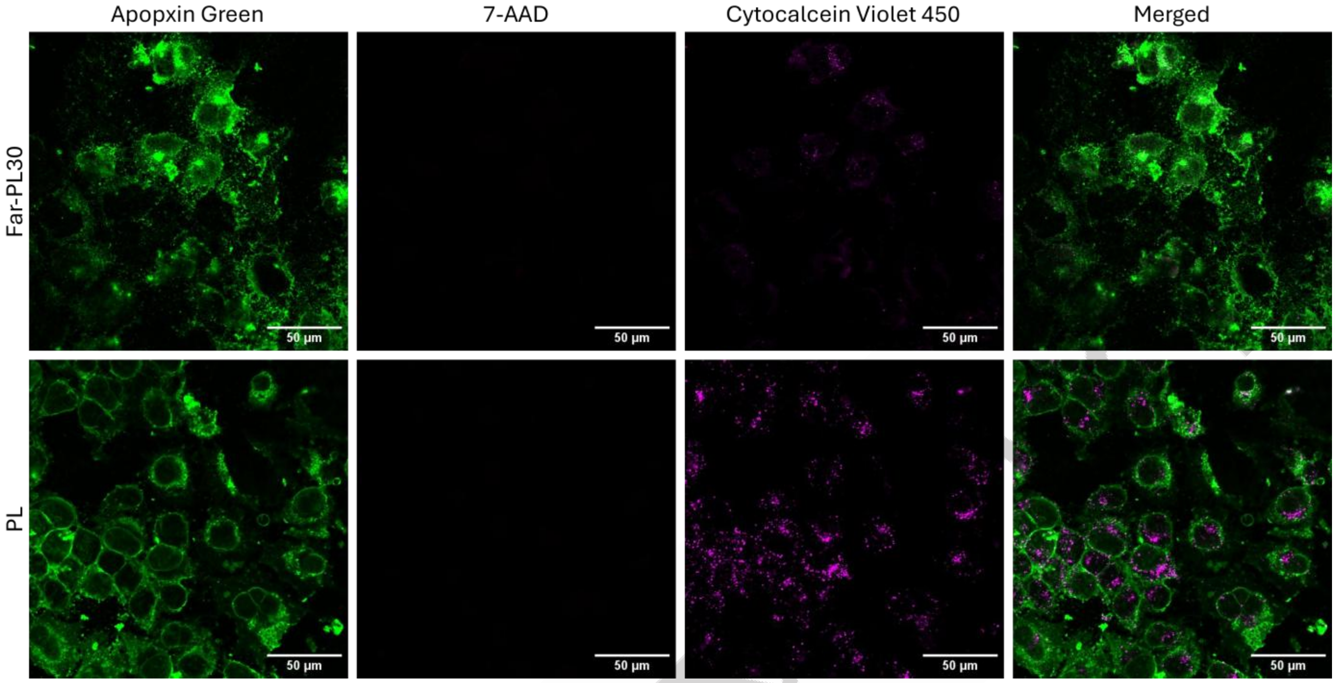

Figure S4 Apoptosis/necrosis assay performed on A549 cells after 2 h incubation with Far-PL30 and PL at a concentration of 62.5 µg/mL. Early apoptotic cells (green), necrotic/late apoptotic cells (red), and viable cells (purple) were stained by Apopxin Green, 7-AAD, and CytoCalcein Violet 450, respectively.

Apopxin Green is a cell marker for apoptotic cells, binding phosphatidylserine (PS) when exposed to the outer membrane during pre-apoptotic events. 7-Amino actinomycin D (7-AAD) is a necrosis marker, negligible in both samples. CytoCalcein Violet 450 is a cell-permeable dye retained by living cells. The image shows apoptosis events for both compounds, with differences in cell viability and morphology. For Far-PL30, the green signal from apoptotic cells is spread among the cells, showing loss of membrane integrity and cell damage, while for PL, the signal is confined to the outer part of the cell membrane. This can be explained by the difference in cytotoxicity between the two compounds. While Far-PL30 presents high cytotoxic activity on cancer cells, leading to cell damage after 2 h (CytoCalcein Violet 450 is hardly retained by cells) and a decrease in cell viability after 24 h (MTT), PL only shows pre-apoptotic events, while cells appear to be still alive and with proper membrane integrity, shown by their ability to retain CytoCalcein Violet 450 and by no cytotoxicity observable after 24 h. From the results, we can conclude that the difference in the two polymers results in different speeds of the apoptotic process, due to the amount of polymer taken up by the cells and the efficacy of the polymer itself.

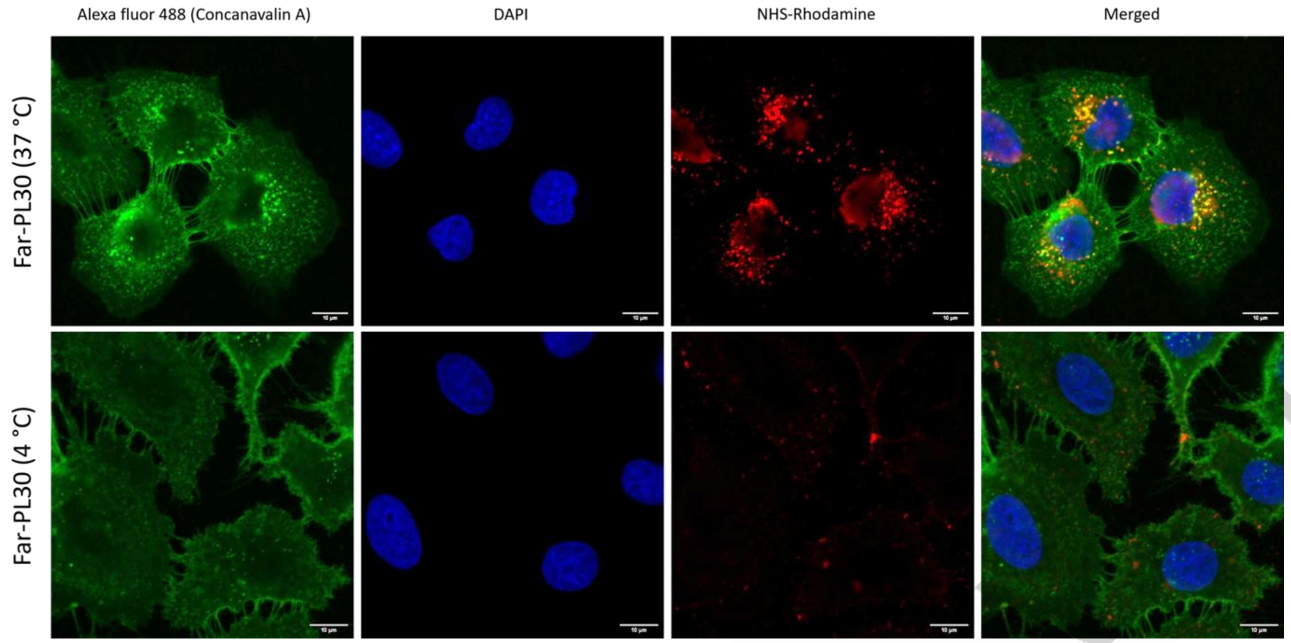

Figure S5 Temperature-dependent uptake of Far-PL30 (10 µg/mL) on A549 cells after 4 h incubation at 4 °C and 37 °C. Far-PL30 showed an increased cellular uptake on A549 at 37 °C compared to 4°C, suggesting an active mechanism of uptake into the cells.

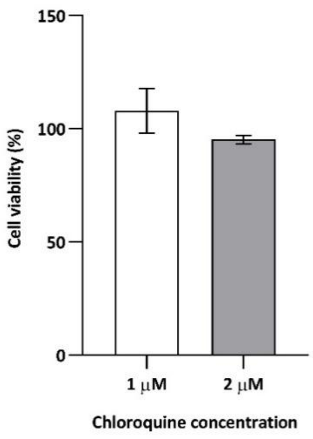

Figure S6 Chloroquine cytotoxicity on A549 after 24 h at the concentration of 1 and 2 µM.
